# Descriptive epidemiology of animal-induced injuries in Sandun Town, Hangzhou, China

**DOI:** 10.3389/fpubh.2025.1680842

**Published:** 2026-01-09

**Authors:** Qilong Wang, Lvzhao Liao, Da Sun, Junfeng Zhao

**Affiliations:** 1 Second School of Clinical Medicine, Zhejiang Chinese Medical University, Hangzhou, Zhejiang, China; 2 Zhejiang Hospital, Affiliated with Zhejiang University School of Medicine, Hangzhou, Zhejiang, China

**Keywords:** animal bite, communicable disease, emergency department, prophylaxis, rabies

## Abstract

**Objective:**

To summarize epidemiological features of animal-induced injury patients using the emergency department medical record system, and provide recommendations for improving animal-induced injury prevention and immunization practices.

**Methods:**

A cross-sectional study was conducted based on data of animal-induced injury patients treated at the Animal Bite Clinic of the Emergency Department of Zhejiang Hospital from January 1 to December 31, 2024.

**Results:**

The study had a median age of 26 years, with the 25–44 year age group being the most represented. Majority of the patients were migrants, and workers constituted the predominant occupational group. Notably, cats were identified as the most common source of injury, followed by dogs, with most incidents occurring indoors. The most frequent clinical presentations were category II wounds and single upper limb injuries. Regarding post-exposure management, although most patients sought treatment on the day of injury, a significant majority had no history of prior rabies vaccination. The completion rate for the full post-exposure vaccination course was high. However, the administration of human rabies immunoglobulin was low, and only a minority of patients received concomitant tetanus vaccine

**Conclusion:**

This study concludes that despite progressive strengthening of immunization programs, China's rabies control efforts require further refinement to bridge the gap with developed countries. The substantial annual burden of animal-induced injuries highlights the necessity for targeted interventions, such as educating specific demographics and emphasizing outdoor vigilance in warm seasons. Improving tetanus vaccination and rabies immunoglobulin administration, alongside consistent patient counseling, are key clinical priorities. The emergency medical record system emerges as a cornerstone for surveillance, offering critical evidence to guide national prevention policies and immunization strategies.

## 1 Introduction

Rabies, an acute zoonotic infectious disease caused by viruses of the Lyssavirus genus, can affect all mammals. Among the lyssaviruses, the rabies virus is the most significant representative and the primary causative agent of human rabies (1–3). Rabies is invariably fatal. Rabies patients initially develop neurological symptoms, with opisthotonos, muscle spasms, and hydrophobia being characteristic manifestations. Once clinical symptoms manifest, the disease is irreversible. Severe cases progress to respiratory muscle spasms, asphyxia, and organ failure, culminating in death (4, 5). However, 99% of rabies cases can be prevented through post-exposure prophylaxis (PEP) (6).

Rabies is present on all continents, except Antarctica, but over 95% of human deaths occur in Asia and Africa. According to the World Health Organization (WHO) statistics, approximately 60,000 individuals die from rabies annually (7). In many developing countries, rabies receives insufficient attention, resulting in high endemicity in economically disadvantaged regions (8). Historically, efforts to combat human rabies yielded limited success until the 19th century, when researchers discovered rabies vaccines and rabies immunoglobulin, enabling effective prevention (9). WHO recommends two prophylactic vaccination regimens: the 5-dose Essen regimen (vaccination on days 0, 3, 7, 14, and 28 post-exposure) and the 4-dose Zagreb regimen (two doses on day 0, followed by single doses on days 7 and 21) (10). Despite advancements in medicine and decreasing vaccine costs, rabies control remains suboptimal.

China is considered a country with a high incidence of rabies, having experienced three major epidemic waves characterized by significant fluctuations: the first in the 1950s, the second from the 1980s to the mid-1990s, and the third between 1996 and 2007. Throughout these periods, the number of animal-induced injury cases remained high (11). Despite increased awareness in recent years, disparities in healthcare access and public awareness across regions has contributed to continued morbidity and mortality from rabies (12), particularly in rural areas (13). Rabies reached an epidemic peak in 2007, with 3,300 reported cases. Subsequently, reported case numbers declined annually. By 2020, reported cases in China decreased to 202. The official enactment of China's Animal Epidemic Prevention Law in 2021 further contributed to control efforts. The release of China's Rabies Prevention Work Guidelines in 2023 has enhanced the standardization of rabies prevention practices nationwide. Nevertheless, more than 120 human rabies cases continue to be reported every year in China. As population of stray and wild animals continues to grow, ongoing surveillance of animal injuries is crucial to support rabies prevention efforts. Recognition of this issue has been growing at the national level, with ongoing public health efforts to raise awareness about the importance of both rabies and tetanus vaccination. Although cases significantly decreased compared to previous years, rabies remain a public health concern, with 120 cases reported nationally in 2023 (14, 15). The 2024 China Pet Industry White Paper indicates that the populations of stray and wild animals in both urban and rural settings have steadily increased (16). This escalating potential for human-animal contact underscores the significance of novel approaches utilizing electronic medical record (EMR) systems for monitoring animal-induced injury patients. The aim of the current study was to describe the epidemiology of animal-induced injuries using hospital emergency department EMR data, to inform improved rabies prevention and immunization strategies.

## 2 Materials and methods

### 2.1 Data source and study design

We conducted a cross-sectional study. Data were collected for animal-induced injury patients who received treatment at the Animal Bite Clinic of the Emergency Department of Zhejiang Hospital between January 1 and December 31, 2024. Clinical data were extracted from both structured fields and free-text clinical notes in the electronic medical record system. Data were extracted from the EMR system by trained physicians using a standardized data collection form. Prior rabies vaccination history was ascertained through a combination of self-reporting by patients and verification against any available historical vaccination records within the regional health information system, where accessible. For variables such as wound grade and exposure risk, the initial assessment documented by the attending physician in the clinical record was taken as the primary reference.

### 2.2 Inclusion criteria included

(1) Patients had no missing clinical data; (2) Patients underwent treatment for an animal-induced injury at the Animal Bite Clinic of the Emergency Department of Zhejiang Hospital; and (3) Patients had no cognitive impairment. Patients with animal-induced injuries were excluded if their history of or planned schedule for post-exposure rabies vaccination could not be verified.

### 2.3 Study variables

These can be grouped into: demographics, injury characteristics, medical interventions and other. The latter regards non-patient information. Data on demographic characteristics were obtained for all patients. This included gender, age, occupation, and residential status (classified as either local or migrant population based on household registration information). We collected detailed information regarding the injury incident. This encompassed the date and location where the injury occurred, the species of the injuring animal, the clinical assessment of the exposure grade and risk level, the anatomical site of the wound, and the number of distinct wounds. Data on prior rabies vaccination history and whether the patient received rabies immunoglobulin or concomitant tetanus vaccination at Zhejiang Hospital were also recorded. The exposure grade was classified according to the WHO-recommended guidelines. Wound risk was categorized as low risk for WHO Category I exposures and high risk for WHO Category II and III exposures. Details of the medical management administered to patients presenting with animal injuries were extracted from the hospital's clinical records. Monthly average temperatures for Hangzhou City, Zhejiang Province, from January 1 to December 31 2024 were obtained from the National Centers for Environmental Information (NCEI), under the National Oceanic and Atmospheric Administration (NOAA). Months were categorized as warm if their monthly average temperature was above 18.8 °C and cold if below.

### 2.4 Statistical analysis

Statistical analysis and graphing were performed using SPSS 26.0 and Prism 9.5.0 software. Continuous data with normal distribution are presented as mean ± standard deviation and compared using Student's *t*-test. Categorical data are presented as percentages (%) and compared using the Chi-square test. Continuous data with non-normal distribution are presented as median (interquartile range) and compared using the Mann-Whitney *U*-test. A multivariate logistic regression model was employed to identify factors independently associated with the completion of the rabies PEP series. The dependent variable was binary with 1 indicating that a patient completed the full rabies vaccination course and 0 otherwise. Explanatory variables included in the model were selected based on their clinical relevance and significant association in univariate analyses. They included wound risk grade, prior rabies immunization history and human rabies immunoglobulin. A *P*-value < 0.05 was considered statistically significant.

### 2.5 Ethical approval

The current study was approved by the Ethics Committee of Zhejiang Hospital (Approval No. ZJHIRB-2025-107K) on June 24, 2025. The requirement for informed consent was waived by the committee due to the retrospective nature of the study and the use of fully anonymized patient data.

## 3 Results

### 3.1 Characteristics of the study population

From January to December 2024, a total of 9,895 patients presented at the emergency department of Zhejiang Hospital in Zhejiang Province. Females predominated (5,501 cases, 55.6%) over males (4,394 cases, 44.4%). The median age was 26 years (range: 4 months to 87 years), with the highest number in the 25–44 year age group. With regard to geographic origin, the majority of cases were from outside the province (5,821 cases, 58.8%), while 4,074 cases (41.2%) were from within the province. The majority of animal-induced injury patients (6,584 cases, 66.5%) had no rabies immunization history prior to the injury (Table 1).

**Table 1:** Characteristics of the animal-induced injury patient population.

**Characteristic**	**Count (%)**
**Gender**	
Male	4,394 (44.4)
Female	5,501 (55.6)
**Age group (years)**	
<1	81 (0.8)
1–6	590 (6.0)
7–14	1,349 (13.6)
15–24	2,241 (22.6)
25–44	4,104 (41.5)
45–64	1,249 (12.6)
≥65	281 (2.8)
**Geographic origin**	
Within province	4,074 (41.2)
Outside province	5,821 (58.8)
**Prior rabies vaccination history**	
Yes	3,311 (33.5)
No	6,584 (66.5)
**Location of injury**	
Outdoor	3,603 (36.4)
Indoor	6,292 (63.6)
**Injuring animal**	
Dog	3,449 (34.9)
Cat	5,932 (59.9)
Rabbit	69 (0.7)
Rodent	410 (4.1)
Other	35 (0.4)
**Categories of exposure (WHO classification)** ^a^	
Category I (clean wound)	672 (6.8)
Category II (unclean/contaminated wound)	6,366 (64.3)
Category III (dirty/infected wound)	2,857 (28.9)
**Wound location**	
Upper limb	7,245 (73.1)
Lower limb	2,009 (20.4)
Head/Face	309 (3.1)
Neck	58 (0.6)
Trunk	274 (2.8)
**Number of wounds**	
Single	9,219 (93.2)
Multiple	676 (6.8)
**Wound management**	
Professional wound care	2,082 (21.0)
Self-care	7,813 (79.0)
2-dose booster	3,311 (33.5)
2-1-1 Zagreb regimen (4-dose)	788 (8.0)
5-dose Essen regimen	5,796 (58.6)
**Receipt of rabies immunoglobulin**	
Received	764 (7.7)
Not received	9,131 (92.3)
**Receipt of rabies immunoglobulin by exposure grade**	
Category I (clean wound)	100 (14.9)
Category II (unclean/contaminated wound)	405 (6.4)
Category III (dirty/infected wound)	259 (9.1)
Concomitant tetanus vaccination (over 11 years old)^b^	1,028 (13.4)

^a^Category I exposure: contact or feeding of animals, or intact skin being licked by animals. Category II exposure: uncovered skin being lightly bitten, or minor scratches/abrasions without apparent bleeding. Category III exposure: single or multiple penetrating bites or scratches; Licking of broken skin; Contamination of open wounds or mucous membranes by saliva or tissue from animals; Direct contact with bats. ^b^The updated rabies guidelines recommend tetanus vaccination for high-risk wounds, specifically WHO category II and III exposures. According to the Non-neonatal Tetanus Guidelines and China's national immunization schedule, children under 11 years of age remain within the protection window of their primary vaccination series and do not require a tetanus booster, even after an animal-induced injury.

### 3.2 Temporal trends

**Figure 1:**
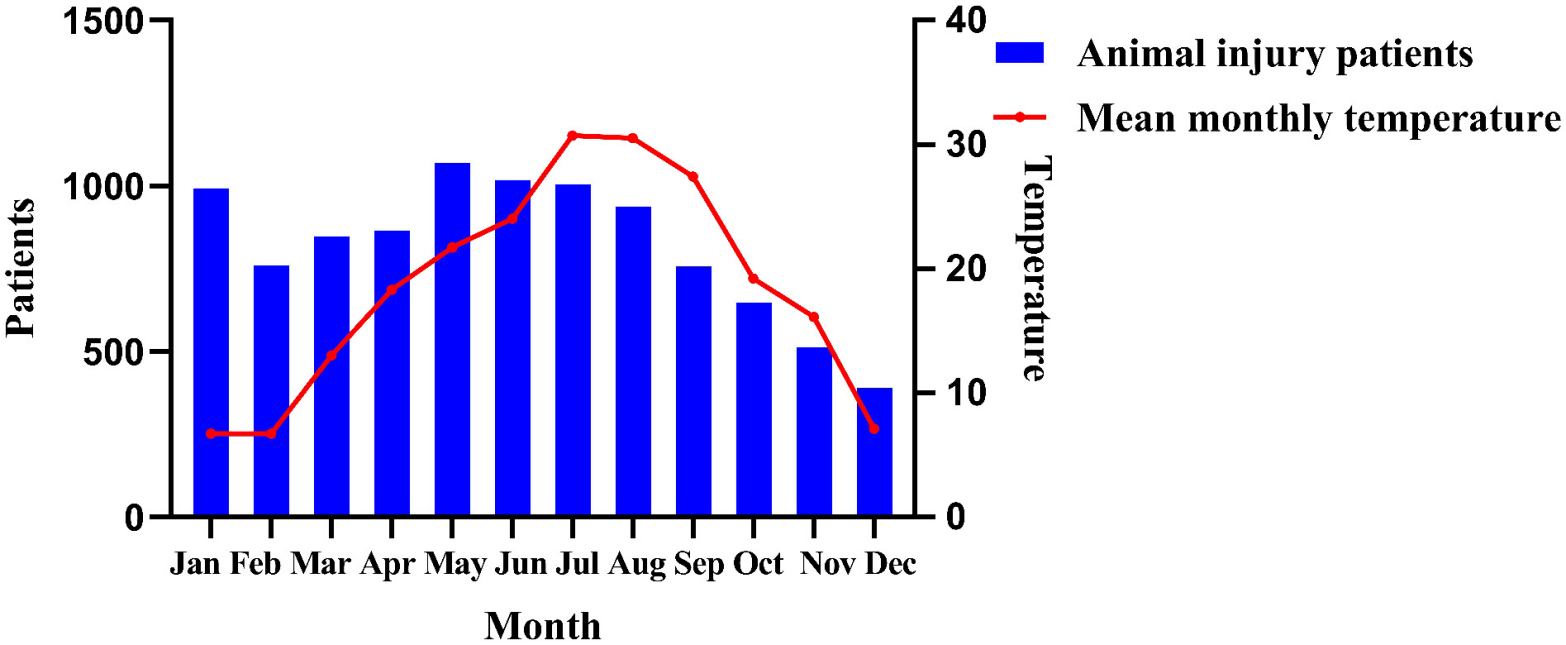
Number of animal-induced injury patients and atmospheric temperature in Hangzhou City, 2024.

From January to December 2024, a high monthly caseload of animal-induced injury patients was observed in the emergency department of Zhejiang Hospital. Animal-induced injury incidents occurred across all months (Figure 1, Table 1).

### 3.3 Injury characteristics and medical management

**Figure 2:**
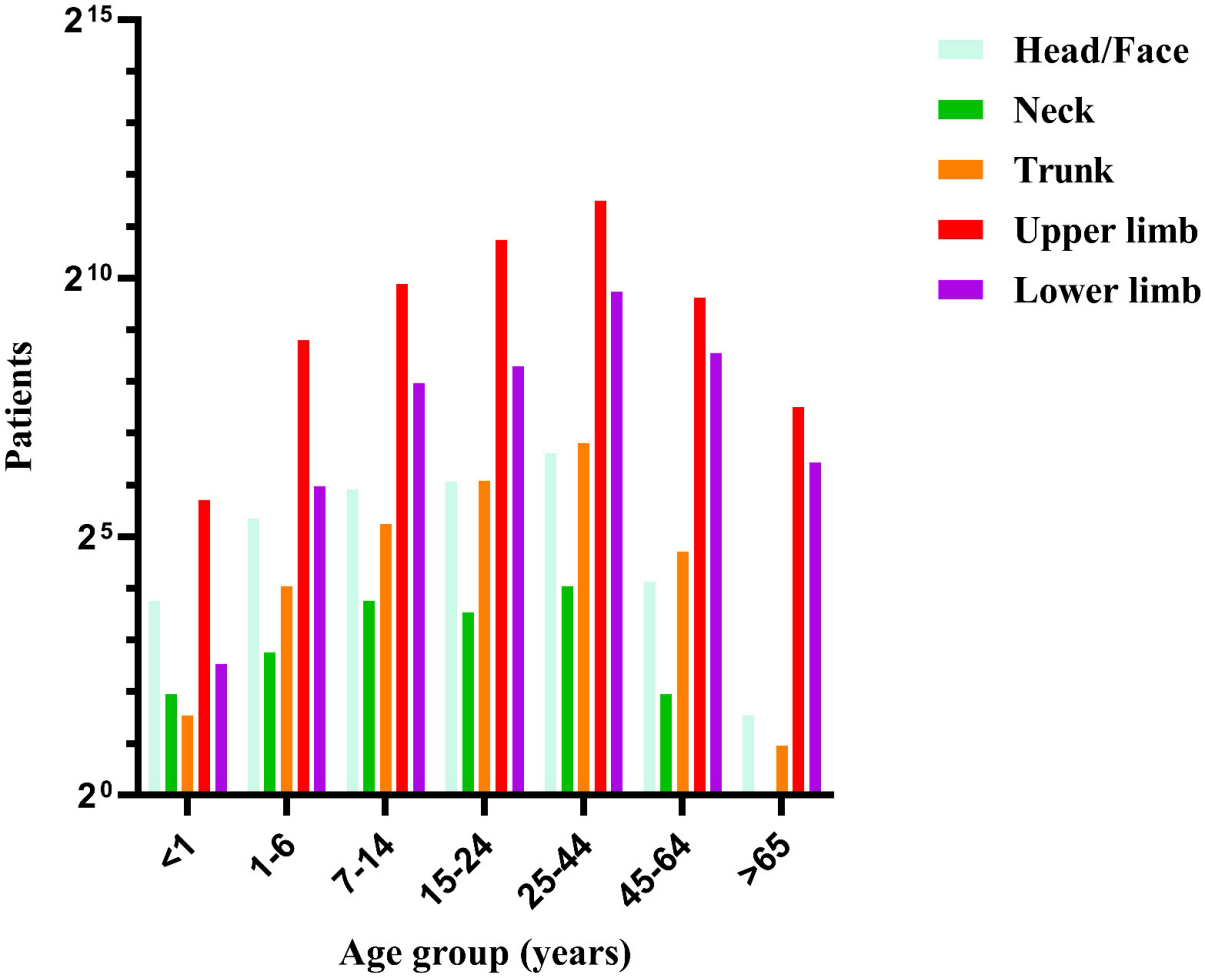
Number of patients by age group and location of injury on body.

**Figure 3:**
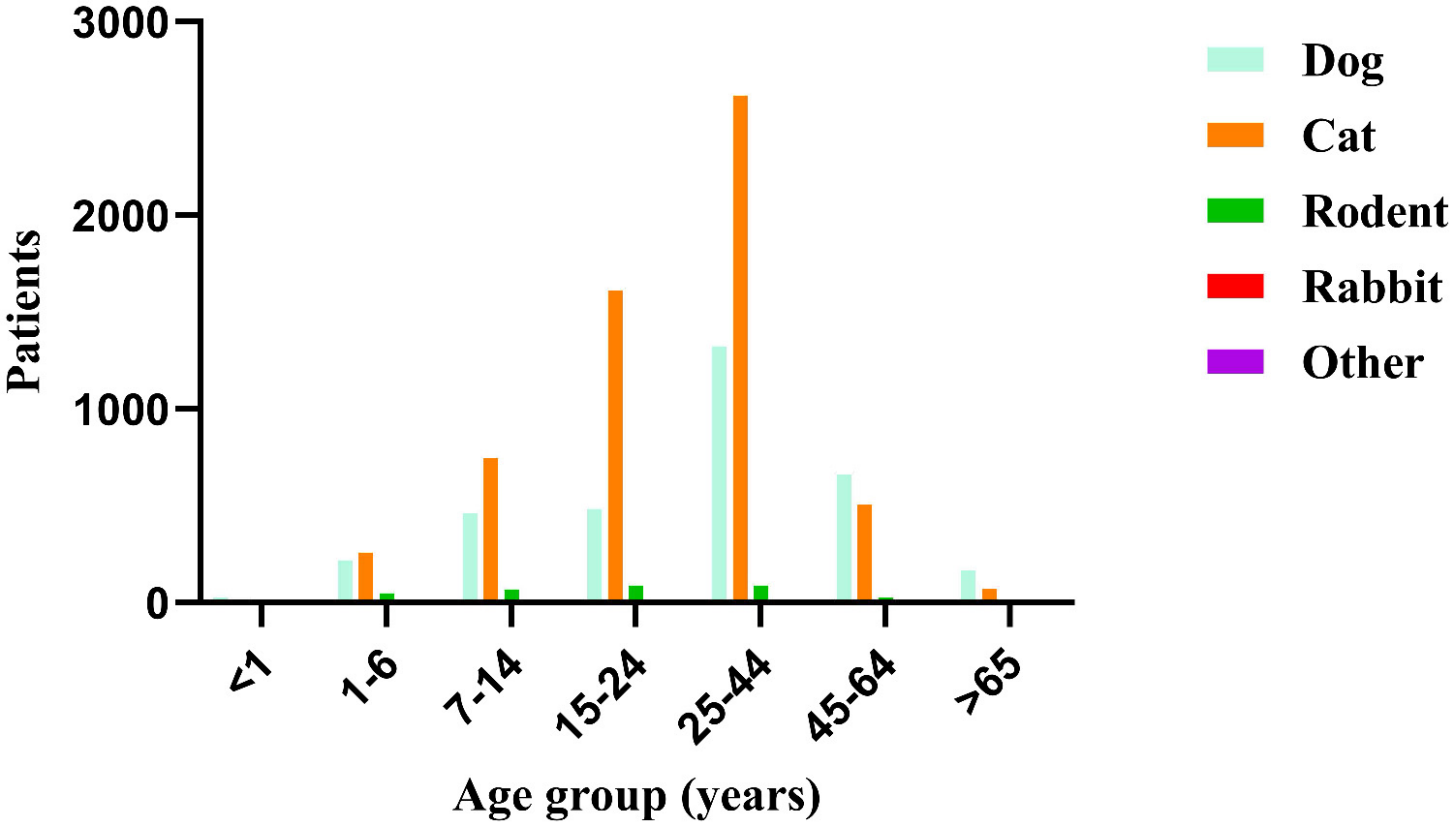
Number of patients by age group and injuring animal type.

**Figure 4:**
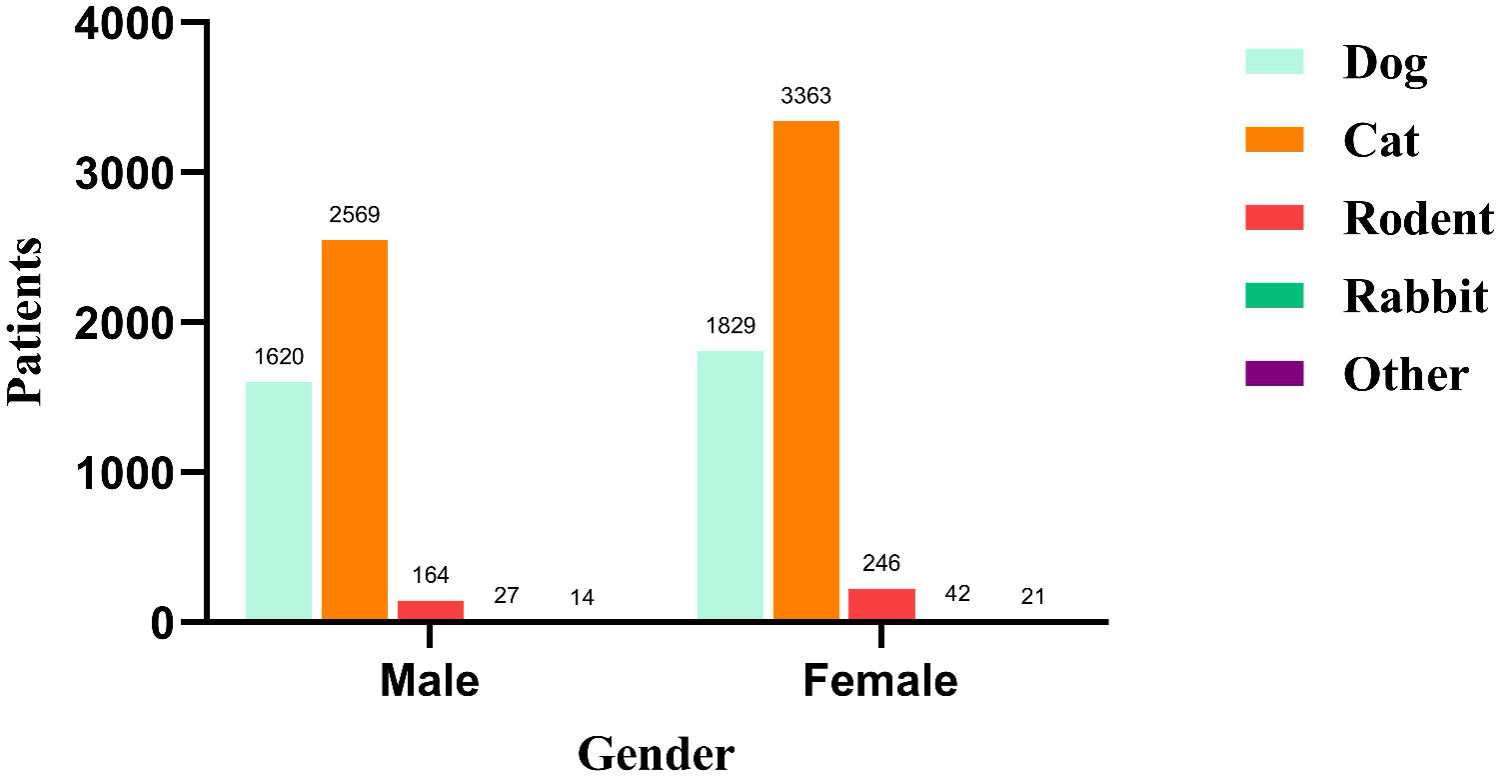
Number of patients by gender and injuring animal type.

No critical cases, rabies infections or deaths were recorded among the 9,895 patients during the study period. Furthermore, no patients required hospital admission for injury-related complications, which served as our criterion for defining a “critically severe case.” The majority of animal-induced injury patients presented to the emergency department of Zhejiang Hospital on the day of injury. The time from injury to consultation ranged from 0 to 254 days. Indoor locations were the primary injury site (6,292 cases, 63.6%). Cats (5,932 cases, 59.9%) and dogs (3,449 cases, 34.9%) were the predominant injuring animals. Category II wounds (contaminated wounds) were the most common wound type (6,366 cases, 64.3%), with single upper limb wounds being predominant. The full post-exposure rabies vaccination course completion rate was 89.6% (8,867 cases). Stratified by age group, animal injuries were more frequent in the 25–44 year age group, with wounds concentrated on the upper limbs. Stratified by sex, females sustained more injuries than males from all animals except dogs. Regarding wound management, most patients performed self-care (7,813 cases, 78.9%). The 5-dose Essen regimen was the primary vaccination schedule (5,796 cases, 58.6%). Only 764 patients (7.7%) received human rabies immunoglobulin. The administration of human rabies immunoglobulin varied by wound category. It was administered to 100 patients (14.9%) with category I wounds, 405 patients (6.4%) with category II wounds, and 259 patients (9.1%) with category III wounds. Concomitant tetanus vaccination was administered to 1,028 patients, which accounted for 13.4% (among 7,667 cases), this figure of 7,667 refers to the subset of animal-induced injury patients who were over 11 years of age and presented with category II or III wounds (Table 1, Figures 2–4).

Outdoor injuries were significantly more likely than indoor injuries during warmer months (^***^P < 0.01). *P*-value was derived from the Pearson χ^2^ test (Table 2).

**Table 2:** Comparison of monthly average temperature and injury event location.

**Characteristic**	**Cold months^a^**	**Warm months**	**χ^2^**	*P*-value^b, ***^
Indoor injury, *n* (%)	3,244 (51.6)	3,048 (48.4)	337.71	<0.01
Outdoor injury, *n* (%)	1,170 (32.5)	2,433 (67.5)		

^a^Cold months and warm months were defined by splitting the study period at the median monthly temperature (18.8 °C), with the 6 warmer months classified as warm months and the 6 cooler months as cold months. ^b^P-value was derived from the Pearson χ^2^ test.

Among pediatric patients aged 14 years and younger, 1,336 (66.1%) had a prior history of rabies vaccination. Regarding the spectrum of injuring animals, cats remained the predominant source, accounting for 1,063 cases (52.6%), followed by dogs in 753 cases (37.3%). Anatomically, the upper limb was the most frequently involved site, comprising 1,488 cases (73.7%), while injuries to the head and face were less common, involving only 118 patients (5.9%). The majority of wounds were classified as category II, totaling 1,415 cases (70.0%) (Table 3).

**Table 3:** Characteristics of animal-induced injuries in patients aged ≤ 14 years.

**Characteristic**	**Count (%)**
**Prior rabies vaccination history**	
Yes	1,336 (66.1)
No	684 (33.9)
**Injuring animal**	
Dog	753 (37.3)
Cat	1,063 (52.6)
Rabbit	11 (0.5)
Rodent	150 (7.4)
Other	43 (2.1)
**Wound location**	
Upper limb	1,488 (73.7)
Lower limb	330 (16.3)
Head/Face	118 (5.9)
Neck	25 (1.2)
Trunk	59 (2.9)
**Categories of exposure (WHO classification)**	
Category I (clean wound)	147 (7.3)
Category II (unclean/contaminated wound)	1,415 (70.0)
Category III (dirty/infected wound)	2,458 (22.7)

Full post-exposure rabies vaccination course completion (adherence) was not associated with patient gender, species of injuring animal, or number of wounds (*P* > 0.05). *P*-value was derived from the Pearson χ^2^ test (Table 4).

**Table 4:** Factors associated with completion of rabies PEP series.

**Characteristic**	**Total (%)**	**Completed vaccination (%)**	**Incomplete vaccination (%)**	**χ^2^**	***P*-value^c^**
**Gender**					
Male	4,394 (44.4)	3,947 (89.8)	447 (10.2)		
Female	5,501 (55.6)	4,920 (89.4)	581 (10.6)	0.40	0.53
**Wound risk grade**					
Low-risk^a^	672 (6.8)	649 (96.6)	23 (3.4)		
High-risk^b^	9,223 (93.2)	8,218 (89.1)	1,005 (10.9)	37.58	<0.01
**Injuring animal**					
Dog	3,449 (34.9)	3,100 (89.9)	349 (10.1)		
Cat	5,932 (59.9)	5,311 (89.5)	621 (10.5)	0.29	0.59
**Number of wounds**					
Single	9,219 (93.2)	8,255 (89.5)	964 (10.5)		
Multiple	676 (6.8)	612 (90.5)	64 (9.5)	0.66	0.41
**Prior rabies immunization history**					
Yes	3,311 (33.5)	3,280 (99.1)	31 (0.9)		
No	6,584 (66.5)	5,587 (84.9)	997 (15.1)	477.61	<0.01
**Human rabies immunoglobulin**					
Received	764 (7.7)	752 (98.4)	12 (1.6)		
Not received	9,131 (92.3)	8,115 (88.9)	1,016 (11.1)	69.16	<0.01

^a^Category I wounds. ^b^Category II/III wounds. ^c^P-values were derived from the Pearson χ^2^ test.

However, it was significantly associated with wound risk grade, prior rabies immunization history, and administration of passive immunizing agents (^*^P < 0.05). *P*-value was derived from the multivariate logistic regression analysis (Table 5).

**Table 5:** Factors associated with completion of rabies post-exposure prophylaxis series: a multivariate logistic regression analysis.

**Characteristic**	**OR**	**95% CI**	***P*-value**
Wound risk grade	4.85	3.17–7.41	<0.01
Prior rabies immunization history	0.04	0.03–0.06	<0.01
Human rabies immunoglobulin	0.08	0.05–0.14	<0.01

## 4 Discussion

In the current study, a total of 9,895 animal-induced injury patients were treated in the emergency department of Zhejiang Hospital between January and December 2024. The majority of the patients were female and concentrated in the 25–44 age group, a demographic profile that corresponded with a high proportion of individuals employed as workers. Most patients had no prior rabies immunization history before injury, this stands in contrast to the pediatric subgroup in this study, among whom 66.1% had a prior history of rabies vaccination, suggesting a potential effect of childhood immunization programs or increased parental vigilance. In terms of gender distribution, occupational composition, and pre-exposure immunization status, these findings are consistent with international studies (17) and largely align with another epidemiological survey on animal-induced injury patients in Hangzhou (18). However, a notable difference was observed in geographic origin. A previous study (18) reported that most animal-induced injury patients were residents of Zhejiang Province, with only a small proportion originating from other provinces. In contrast, the current study found that the majority of patients were from outside Zhejiang Province. This discrepancy may be attributed to the demographic composition of the population served by the study hospital. Hangzhou, as a relatively developed urban center, has a large migrant worker population compared to rural areas. Additionally, since the issuance of China's Rabies Prevention Guidelines in 2023, tetanus vaccination advocacy and standardized management of animal-related injuries have been widely promoted. Consequently, during the data collection period of the current study, a considerable proportion of non-local residents received standardized post-exposure care. Demographic patterns reported in other regions of China vary, suggesting distinct epidemiological characteristics of animal injuries across different geographic settings. In summary, based on the demographic profile identified, animal-induced injury prevention education should primarily target inter-provincial migrant workers aged 25–44 years.

Regarding injury characteristics, the majority of animal-induced injury patients presented to the emergency department of Zhejiang Hospital on the day of injury. The primary injury location was indoor (63.6%), and cats were the predominant injuring animals (59.9%). This finding differs from reports both domestically and internationally, where dogs are typically predominant (19–22). Combined with the high proportion of indoor injuries (63.6%) and upper limb wounds (73.1%) in the current study, it is plausible that injuries were largely inflicted by household pets. A possible explanation for the predominance of cat injuries could be a cultural preference for keeping cats as pets over dogs in urban Chinese households. This companionship may explain why indoor incidents are most predominant. This pattern is also markedly different from international reports (23, 24), potentially reflecting regional differences. Dogs in China are more frequently kept as outdoor pets, particularly in rural settings, which may contribute to different exposure patterns. Globally, dogs represent the primary source of animal-related injuries. Consistent with existing literature, children most frequently sustain dog bites to the head and neck regions, whereas adolescents and adults are more commonly injured in the extremities and hands. Dog bites often result in crush injuries and soft tissue avulsions, while cat bites typically cause deeper puncture wounds (25). Consequently, imaging studies should be considered for patients with dog bites to assess underlying tissue damage. For cat-induced puncture wounds, careful evaluation of wound depth is essential; thorough debridement should be performed to prevent pocket formation, and tetanus vaccination must be ensured in such cases. Patients who received human rabies immunoglobulin or had a prior rabies vaccination history demonstrated higher adherence to medical advice, exemplified by the higher rate of full rabies vaccination course completion. This aligns with domestic research (26). Paradoxically, however, patients with lower-risk wounds in the current study showed higher adherence than those with higher-risk wounds. This discrepancy may occur for two reasons. First, patients with high-risk wounds were likely preoccupied with immediate care, such as wound suturing. This urgency may have led them to underestimate the importance of completing the vaccination series. Alternatively, patients with lower-risk wounds may have been more likely to have a prior rabies immunization history, which is associated with better adherence. And in the current study, we discovered a case like this: a patient presented to the emergency department 254 days after the animal exposure. Upon inquiry, the patient reported that they decided to seek care after encountering educational materials about rabies prevention. Notably, no typical symptoms of rabies had developed during this prolonged interval. Following China's Rabies Prevention Work Guidelines (2023), the patient was administered the rabies vaccine upon presentation.

Children and infants represent a high-risk group for animal-related injuries due to their innate curiosity. Numerous international studies indicate that children are particularly vulnerable to animal attacks, especially from dogs, and sustain a higher frequency of injuries to the head, face, and neck (27, 28), often resulting in severe cases requiring hospitalization. However, our findings diverge from this pattern. Similar to the adult group in this study, the most frequently injured anatomical region in pediatric patients was the upper limbs, with the majority of wounds classified as category II. Furthermore, cats—not dogs—were identified as the primary injuring animals. This distinctive profile may be attributed to the urban setting of our study, where children are more likely to encounter household pets than stray animals, which are less prevalent in urban compared to rural environments. Consequently, injuries predominantly result from interactive contact, typically involving the upper limbs. Additionally, the presence of a dedicated pediatric hospital in the region may have diverted the most severe pediatric cases away from the general emergency department of Zhejiang Hospital, potentially contributing to the observed injury characteristics in this study.

Correlating with the 2024 monthly average temperatures in Hangzhou, Zhejiang Province, outdoor injuries were significantly more likely during warmer months compared to indoor injuries (^***^P < 0.01). This seasonal variation is potentially attributable to increased outdoor activities with greater skin exposure during warm weather and heightened activity levels among stray animals. This observation highlights the need for heightened vigilance against outdoor animal injuries, particularly from cats, during warmer months. Reinforced education is especially crucial for patients with no prior rabies immunization history and high-risk wounds.

It is noteworthy that the utilization rate of human rabies immunoglobulin in the current study was low (7.7%). According to the 2023 Chinese Rabies Prevention Guidelines, human rabies immunoglobulin is recommended for all patients with WHO category III exposure, in addition to rabies vaccine. However, among such patients in this study, the human rabies immunoglobulin administration rate was only 26.7%, which is lower than that reported in other domestic animal-induced injury studies (19). Multiple factors may contribute to patients' reluctance to receiving human rabies immunoglobulin. First, the high cost of the therapy deters many individuals, who often opt for rabies vaccine alone. In China, rabies PEP, including immunoglobulin, is often an out-of-pocket expense. While basic medical insurance may cover a portion of the cost in some regions, reimbursement rates for such biologics are typically low, leaving patients to bear the majority of the financial burden. Second, there may be insufficient awareness of the updated guidelines among clinicians. It is noteworthy that rabies immunoglobulin was administered even in cases of category I clean wounds. While partly attributable to patients' strong requests, this practice underscores a gap in knowledge regarding the updated guidelines among some clinicians. Third, there is generally low risk perception of rabies among exposed populations, coupled with limited access to targeted health education and a tendency to underestimate potential risks. Fourth, a previous domestic study suggested that individuals above 10 years of age are less likely to receive human rabies immunoglobulin due to factors such as population mobility, low protection awareness, non-standard practices, and irregular vaccination record management (29). This finding highlights a critical gap in the current rabies prevention system—specifically, the under implementation of human rabies immunoglobulin—and underscores the need for administrative and educational interventions to improve adherence to guidelines.

Additionally, within the current study, 6,584 patients (66.5%) had no prior history of rabies vaccination. International research recommends that adults complete recommended vaccination courses (30), including post-exposure rabies vaccination when seeking PEP. Utilizing emergency electronic medical records and other rabies/animal-induced injury surveillance systems, China could optimize post-exposure rabies vaccination protocols, potentially including them in national immunization programs.

Recent studies and reports emphasize the necessity for appropriate medical management of animal-induced injury wounds, including anti-infection treatment and essential ancillary examinations, with tetanus prophylaxis gaining increased attention (31, 32). However, the current study reveal that only a minority of patients (10.4%) received tetanus vaccination. This low rate can be partially explained by national guidelines. According to China's 2024 Non-neonatal Tetanus Diagnosis and Treatment Guidelines, individuals under 11 years of age are considered protected and do not require tetanus vaccination following an injury (33, 34). Even after excluding this group, a substantial proportion of eligible patients remained unvaccinated. Key factors contributing to low tetanus vaccine uptake among animal-induced injury patients include inconsistent adherence to updated guidelines among surgeons and low patient compliance/perceived necessity. This necessitates mandatory training for emergency department surgeons and enhanced public education.

Strengths of the current study include: (1) The hospital from which the data were sourced is one of the first in Zhejiang Province to administer tetanus vaccination, providing a large sample size of animal-induced injury patients. The data were derived from the hospital's emergency electronic medical record system, enhancing reliability; (2) As previously indicated, tetanus vaccination for animal-induced injury patients has gained attention in China only in recent years, whereas previously only post-exposure rabies vaccination was emphasized. Thus, the data presented in the current study are relatively novel; and (3) The ability to track completion of the full rabies PEP course, a metric often unavailable in studies that are limited to data from a single emergency visit.

Limitations of the current study include: (1) The data were obtained from the emergency department of a single hospital, serving a population of roughly 100,000 people. Therefore, the findings may not be fully representative of animal-induced injury patterns across China; (2) As a cross-sectional descriptive study, this research cannot establish causal relationships, nor can it identify all factors associated with animal-induced injuries; and (3) Due to the unavailability of official age- and gender-stratified population data for the precise catchment area of the emergency department of Zhejiang Hospital, we were unable to perform population standardization adjustments in the current study. Consequently, the reported case distribution may have been influenced by the underlying demographic composition of the local population. Future research will involve expanding the scope of data collection and implementing long-term follow-up studies on animal-induced injury patients. This approach aims to generate more precise data to better inform prevention and immunization practices.

In summary, while human rabies prevention and control in China have improved by progressively strengthening human immunization programs, a disparity compared to developed nations. The high annual caseload of animal-induced injury patients necessitates enhanced public health education, particularly targeting students, migrant workers, and pet owners (especially those with household cats). Intervention efforts should focus on warmer months (summer and autumn) and emphasize vigilance in outdoor settings, with specific reinforcement of tetanus prophylaxis education for animal-induced injury patients. For healthcare providers, repeated counseling and education are essential for patients with no prior rabies immunization history and high-risk wounds. The emergency medical record system represents a highly effective surveillance tool, providing valuable data to inform strategy development and public awareness campaigns by prevention and immunization authorities.

## Data Availability

The original contributions presented in the study are included in the article/supplementary material, further inquiries can be directed to the corresponding author.
